# Editorial: Insights in Public Mental Health: 2021

**DOI:** 10.3389/fpubh.2022.935922

**Published:** 2022-06-08

**Authors:** Wulf Rössler

**Affiliations:** Department of Psychiatry and Psychotherapy, Charité Universitätsmedizin Berlin, Berlin, Germany

**Keywords:** Public Mental Health, mental health care, healthcare workers, psychiatric epidemiology, systematic review, non-pharmacological interventions, stigma

We are now entering the third decade of the twenty-first century, and, especially in the last years, the achievements made by scientists have been exceptional, leading to major advancements in the fast-growing field of Public Mental Health.

Mental health must be considered a dynamic state, whereby individual psychosocial development is influenced by multiple layers of intersecting social and environmental factors. Good mental health enables individuals to work, to learn, to engage with other people and to participate in society. Conversely, mental disorders cause significant suffering for individuals, their families and society as a whole. Mental disorders arise early in life's course and may last for long periods of time. Persons suffering from mental disorders, have a significantly reduced life expectancy. Public mental health aims to improve the mental health of the population by providing information about levels of mental disorders across populations and also by preventing mental disorders and promoting mental health. Public Mental Health also takes responsibility for the provision of mental health services. As such Public Mental Health aims to publish major insights into the promotion of mental health, the prevention of mental disorders, as well as the provision of care.

This Research Topic “Insights in Public Mental Health: 2021” is focused on new insights, novel developments, current challenges, latest discoveries, recent advances and future perspectives in the field of Public Mental Health. The goal of this Research Topic is to shed light on the progress made in the past decade in the Public Mental Health field and on its future challenges to provide a thorough overview of the status of the art of the Public Mental Health field. This article collection will inspire, inform and provide direction and guidance to researchers in the field.

In recent years, psychiatric genetic research has gained a lot of public attention, but in fact the majority of mental problems arise from social and physical environmental influences. Mental health is a dynamic state, where the development of an individual is influenced by multiple layers of intersecting social and environmental factors. To facilitate our current understanding of Public Mental Health, Kousoulis and Goldie are proposing a new visualization for the traditional socio-ecological model approach.

Psychiatric epidemiology is a cornerstone of Public Mental Health. Nonetheless data about the frequency and distribution of mental disorder are rare. In China, some studies were conducted to assess the prevalence of mental disorders at the national level and in some metropolitan cities. However, the prevalence of mental disorders in Chinese underdeveloped provinces has not been reported internationally in recent decades (Sun et al.). Rates assessed In this study were comparably low and warrant further confirmation.

Differences in rates of mental disorders between women and men have been known for a long time. For most internalizing disorders (e.g., major depression and eating disorders), women are more frequently affected, whereas for externalizing disorders (e.g., substance abuse) more men are affected. This systematic review takes a closer look at similarities and differences of mental health in women and men from three large German epidemiological cohorts (Otten et al.).

The association between social capital and depression is a frequent Research Topic in developed countries, often with inconclusive results. Furthermore, for both social capital and depression, there are gender differences established in the literature. This study from Brazil investigates gender differences in the association of social capital with the incidence and maintenance of depressive episodes (Souto et al.). The results highlight the importance of the dimension “social support” in both genders in its association with mental health.

Research on the association between food insecurity and bullying victimization is limited. Using a representative global sample, this study aimed to investigate the association between food insecurity and bullying victimization in adolescents and whether the association varied between country income levels, sexes, and age groups (Liang et al.). Bullying victimization is prevalent among global adolescents with food insecurity being a significant correlate.

To monitor population mental health, the identification of relevant indicators is pivotal. This scoping review provides a comprehensive overview of current indicators representing the various fields of public mental health core topics (Peitz et al.). It was conducted as a first step to build up a Mental Health Surveillance for Germany.

The majority of people who die by suicide have never seen a mental health professional or been diagnosed with a mental illness. To date, this majority group has largely been ignored, with most existing research focusing on predictors of suicide such as past suicide attempts. Identifying the characteristics of people who die by suicide without receiving services, often with a fatal first attempt, is crucial to reduce suicide rates through guiding improvements to service pathways and “just in time” interventions. In this systematic review the current state of the art is reported (Tang et al.).

Public interest for mental health services mostly focus on industrialized or first world countries, less so on developing countries. This narrative synthesis and perspective piece encompasses an overview of mental health care competencies, best practices and capacity building needed to fast track patient responsive services in Kenya and gives us an idea what is needed to improve mental health care in an African country (Kumar et al.).

During the last decade “air pollution” was found to be related to mental health problems. Two studies deal with this issue. One study examines the heterogeneous impact and mediating mechanisms of air pollution on mental health based on data of 51 countries from 2010 to 2017 The findings show that there is heterogeneous impact of air pollution on different types of mental health. Specifically, air pollution has a significant positive impact on depression (Hu et al.). Researchers from Taiwan investigated this question in depth, analyzing sample of older adults was recruited from a nationally representative multiple-wave study, the Taiwan Longitudinal Study on Aging. They confirmed that exposure to traffic-associated air pollutants could increase depression risks among older adults (Wang et al.).

What does social support sound like? Passive audio data collection on mobile phones (e.g., episodic recording of the auditory environment without requiring any active input from the phone user) enables new opportunities to understand the social environment (Poudyal et al.). Passively capturing audio obviously has the potential to improve understanding of the social environment. This is a good example how creative research in public mental health can be.

Another neglected aspect of our social environment is art and cultural participation. Many different forms of art and cultural participation, for example, visiting museums and galleries, have received increasing attention as an important new focus for public health. The purpose of this study was to explore the effects of art and cultural participation of museums and galleries on life satisfaction intervened and controlled by physical and mental health and interpersonal relationships and individual's background (Lee et al.). It seems that art and cultural participation had direct effects on life satisfaction and indirect effects through the development of interpersonal relationships.

Job-related distress has been in the focus of public mental health research for decades, best known under the name "burnout.” Job-related distress has a well-documented health-damaging and life-threatening character. In this article, Schonfeld and Bianchi review recent developments in research on job-related distress and examine ongoing changes in how job-related distress is conceptualized and assessed. They argue, that the construct of burnout have proved to be problematic and propose to address job-related distress within the long-established framework of depression research.

Job-related distress is not equally distributed across all occupational groups. Employees in the healthcare sector, for example, are particularly exposed to such stress. The COVID-19 pandemic has had an additional impact on the mental health and wellbeing of health workers on the frontlines of the pandemic. The purpose of this article is to provide an evidence-based overview of the adverse mental health impacts on healthcare workers during times of crisis and other challenging working conditions and to highlight the importance of prioritizing and protecting the mental health and wellbeing of the healthcare workforce, particularly in the context of the COVID-19 pandemic (Søvold et al.).

One pivotal component of health care is humanized care because the professional work of nursing seeks to provide quality services to patients who are suffering and fear illness. The main objective of this work was to find scientific evidence on humanized care from the perspectives of nurses and hospitalized patients in a exploratory systematic review (Meneses-La-Riva et al.).

Life transitions are often periods of increased risks for mental health problems. One of these risk period is the transition from home, school or college into university. Although pressure to perform academically whilst fulfilling the stereotypical student life is keenly felt during the transition period, many students conceal their struggles from family and friends (Worsley et al.).

As the majority of mental disorders have an onset before the age of 18, it is a point of major interest, what causes adolescents' emotional distress. Results from this qualitative study show that distinct categories for perceived cause of emotional distress exist (e.g., perceived lack of control or unfair treatment) among adolescents considered to be “at risk” of developing mental health difficulties (O'Neill et al.).

COVID-19 physical distancing measures had a detrimental effect on adolescents' mental health. Adolescents worldwide alleviated the negative experiences of social distancing by spending more time on digital devices. The key message of this systematic review is that not all uses of digital media had negative consequences on adolescents' mental health during the pandemic. Their results suggested that social media use was helpful in mitigating the feeling of loneliness during COVID-19, but only when a one-to-one or one-to-few communication, rather than a general social media use, was promoted (Marciano et al.).

The global incidence of premenstrual syndrome and premenstrual dysphoric disorder is increasing, with increasing suicide reports. However, the bibliometric analysis of global research on these disorders is rare. This bibliometric analysis aimed to evaluate the global scientific output of research on PMS and PMDD and to explore their research hotspots and frontiers from 1945 to 2018 (Gao et al.). Keyword and reference analysis indicated that the menstrual cycle, depression and ovarian hormones were the research hotspots, whereas prevalence, systematic review, anxiety and depression and young women were the research frontiers.

The sport community has paid increased attention to the mental health of elite athletes. While sports participation can bring lots of benefits to elite athletes, they usually need to cope with demands, e.g., career dissatisfaction, performance, expectation, and sport injuries, which can pose potential threats to their mental health. The aim of this cross-sectional study was to examine the prevalence of Generalized Anxiety Disorder (GAD) and its risk and protective factors in elite collegiate athletes (Li et al.). The overall prevalence of GAD symptoms was 22%. Athletes with a history of sport injury, a high risk of ADHD, and a high level of fear of failure had a significant and positive association with GAD, while high levels of mental toughness and satisfaction in sport were significantly and negatively related to GAD.

Non-pharmacological interventions for mental health problems are always of Interest for public mental health practice. As such, the World Health Organization (WHO) has encouraged the inclusion, recognition, and use of Integrative and Complementary Health Practices in national health systems. This evidence map presents a summary of studies that addressed the effects of meditation on various clinical and health conditions (Schlechta Portella et al.). Several meditation techniques were evaluated in different contexts. Most of the studies reported positive effects and a beneficial potential of the practice of meditation.

Another non-pharmacological treatment offered as psychological support is bibliotherapy, which can be described as the process of reading, reflecting, and discussing literature to further a cognitive shift. The COVID-19 pandemic demands a response to prevent a collapse of mental health services. Thus, this study aimed to review articles on the effectiveness of bibliotherapy on different mental health problems (Monroy-Fraustro et al.). This mixed-methods systematic review showed, that through bibliotherapy patients developed new coping capacities. Additionally, values such as autonomy and justice were closely linked with positive results in bibliotherapy.

Another focus of public mental health research is the link between nutrition and mental health, as recent studies suggested that nutritional supplementation may be beneficial in improving mental disorders. This systematic review meta-analysis examined the effectiveness of polyphenol supplementation, which may have anti-inflammatory and antioxidant properties in improving depression, anxiety and quality of life (Lin et al.). The results suggest that polyphenol supplementation is effective in improving depression.

Last not least there is one overarching topic in the field of public mental health: the stigma of mental disorders. This study investigated factors affecting gender-specific stigmatization in the context of drug addiction (Sattler et al.). Their results showed higher stigma if the person used “harder” drugs, displayed aggressive behavior, or had a less controllable drug urge. Knowledge about addiction or prior drug use increased some forms of stigma, but reduced others.

Studies presented here only represent a small sample of the large number of published studies in Public Mental Health 2021 in the section Public Mental Health of Frontiers Public Health, Frontiers Psychiatry and Frontiers Sociology. Many others would have been worth mentioning here as well. Public Mental health has a vibrant research community and submissions in 2022 show a further increasing interest in the field. It is to be hoped that the many interesting scientific results will also be reflected in mental health care worldwide.

## Author Contributions

The author confirms being the sole contributor of this work and has approved it for publication.

## Conflict of Interest

The author declares that the research was conducted in the absence of any commercial or financial relationships that could be construed as a potential conflict of interest.

## Publisher's Note

All claims expressed in this article are solely those of the authors and do not necessarily represent those of their affiliated organizations, or those of the publisher, the editors and the reviewers. Any product that may be evaluated in this article, or claim that may be made by its manufacturer, is not guaranteed or endorsed by the publisher.

